# *clag9* Is Not Essential for PfEMP1 Surface Expression in Non-Cytoadherent *Plasmodium falciparum* Parasites with a Chromosome 9 Deletion

**DOI:** 10.1371/journal.pone.0029039

**Published:** 2011-12-19

**Authors:** Adéla Nacer, Emeric Roux, Sébastien Pomel, Christine Scheidig-Benatar, Hiroshi Sakamoto, Frank Lafont, Artur Scherf, Denise Mattei

**Affiliations:** 1 Institut Pasteur, Biology of Parasite-Host Interactions Unit, Department of Parasitology and Mycology, Paris, France; 2 CNRS URA2581, Paris, France; 3 Cellular Microbiology of Infectious Pathogens, Institut Pasteur de Lille, Lille, France; 4 CNRS UMR8204, Lille, France; 5 Inserm U1019, Lille, France; 6 Université Lille Nord de France, Lille, France; Weill Cornell Medical College, United States of America

## Abstract

**Background:**

The expression of the clonally variant virulence factor PfEMP1 mediates the sequestration of *Plasmodium falciparum* infected erythrocytes in the host vasculature and contributes to chronic infection. Non-cytoadherent parasites with a chromosome 9 deletion lack *clag9*, a gene linked to cytoadhesion in previous studies. Here we present new *clag9* data that challenge this view and show that surface the non-cytoadherence phenotype is linked to the expression of a non-functional PfEMP1.

**Methodology/Principal Findings:**

Loss of adhesion in *P. falciparum* D10, a parasite line with a large chromosome 9 deletion, was investigated. Surface iodination analysis of non-cytoadherent D10 parasites and COS-7 surface expression of the CD36-binding PfEMP1 CIDR1α domain were performed and showed that these parasites express an unusual trypsin-resistant, non-functional PfEMP1 at the erythrocyte surface. However, the CIDR1α domain of this *var* gene expressed in COS-7 cells showed strong binding to CD36. Atomic Force Microscopy showed a slightly modified D10 knob morphology compared to adherent parasites. Trafficking of PfEMP1 and KAHRP remained functional in D10. We link the non-cytoadherence phenotype to a chromosome 9 breakage and healing event resulting in the loss of 25 subtelomeric genes including *clag9*. In contrast to previous studies, knockout of the *clag9* gene from 3D7 did not interfere with parasite adhesion to CD36.

**Conclusions/Significance:**

Our data show the surface expression of non-functional PfEMP1 in D10 strongly indicating that genes other than *clag9* deleted from chromosome 9 are involved in this virulence process possibly *via* post-translational modifications.

## Introduction

An important factor contributing to the virulence of *Plasmodium falciparum* is the ability of parasitized red blood cells (PRBC) to adhere to receptors such as CD36 or ICAM-1 expressed on the surface of endothelial cells, or chondroitin sulphate A (CSA) expressed on placental syncytiotrophoblasts (see [1] for a review). PRBC sequestration can lead to complete blockage of the microvasculature resulting in severe disease including cerebral malaria that may be fatal [2].

Parasite survival within the newly invaded erythrocyte depends on the synthesis and export of several components, which will be used to build the trafficking pathway and channels necessary for the import and export of essential constituents (see [3]). The variant antigen *P. falciparum* erythrocyte membrane protein 1 (PfEMP1) is the predominant ligand responsible for adhesion to host endothelial receptors and is essential for parasite survival and establishing chronic infection [2]. Approximately 60 *var* genes of the parasite's haploid genome encode for PfEMP1 and their expression occurs in a mutually exclusive manner. The *var* genes share a two exon structure with exon 1 coding for the extracellular highly variable adhesive region and a conserved cytoplasmic exon 2 that codes for a transmembrane region (TM) and interacts with the red blood cell cytoskeleton [4]. Exon 1 is composed of variable numbers of Duffy binding-like domains (DBL) and Cysteine-Rich Interdomain Regions (CIDR) that specifically bind to the different receptors in adhesion assays when expressed in heterologous expression systems [5]–[7].

During *in vitro* culture, *var* genes are transcribed and translated in ring stages and, despite the absence of an N-terminal signal sequence [8], PfEMP1 is exported through the endoplasmic reticulum pathway, and beyond the parasite's confines to the erythrocyte surface *via* the Maurer's clefts by interacting with the structural component Knob-Associated Histidine-Rich Protein (KAHRP) [9]. In general, a single *var* gene is expressed in a parasite, but expression can switch to another member in the absence of an immune pressure, leading to antigenic and phenotypic variation at the PRBC surface [10]. This is believed to drive escape from the host's immune response and is implicated in pathogenesis.

A recent large-scale knockout study highlighted the complexity of the interaction between parasite proteins secreted into the RBC cytoplasm and cytoadhesion [11]. In this work and other studies several proteins were identified that contribute either in loading PfEMP1 into Maurer's clefts [11] or transfer from the clefts to the erythrocyte surface [11]–[13]. Although this fascinating mechanism is being intensely studied, many cellular processes involved in trafficking of parasite proteins into the host cell remain elusive [3], [14]. In this work we identified *P. falciparum* laboratory lines that have irreversibly lost their adhesive properties but express non-functional and trypsin-resistant PfEMP1 molecules on the surface of PRBC. Furthermore, to determine if loss of cytoadherence may have resulted from the absence of the cytoadherence-linked asexual gene (*clag9*) due to the truncation of chromosome 9 in D10, we performed targeted disruption of *clag9* and show, that in contrast to previous studies [15], [16], this gene is not essential for the cytoadhesion of PfEMP1.

## Methods

### Parasites and cell cultures

*Plasmodium falciparum* D10, a cloned line derived from FC27 [17], FCR3 [18], Malayan Camp (MC) [19] and 3D7 [20] were cultured as described [21]. All parasite strains were systematically selected for the presence of knobs by gelatine flotation (Plasmion, Fresenius Kabi, France) [22]. Parasite cultures were synchronized by sorbitol lysis [23] and verified for the absence of mycoplasma that could interfere with cytoadhesion [24]. Amelanotic C32 melanoma cells [25] and COS-7 cells [6] were grown in RPMI 1640 without glutamine (Invitrogen), supplemented with 10% foetal bovine serum (heat-inactivated), 0.4 mM L-glutamine, 20 mM HEPES and penicillin/streptomycin.

### Nucleic acids extraction and analysis

Total RNA from 3D7, D10 and FCR3 was isolated by the addition of TRIzol (Invitrogen) to the harvested ring-, trophozoite- and schizont-PRBC [26], processed as described [27], transferred onto Hybond N+ (Amersham) and hybridized with ^32^P-labelled DBL1α or ATS (Acidic Terminal Segment) probes.

Cultured parasites were permeabilized with 0.15% saponin [28] before extraction of genomic DNA (GenElute Mammalian Genomic DNA miniprep kit, Sigma). The full PfEMP1 gene sequence was obtained by PCR amplification and determined by gene walking (Supplementary Table 1), and is available from Genbank (accession number JF342594).

For Southern blotting genomic DNA was digested with *EcoRI* and *SpeI* (BioLabs), and processed as described [29]. Northern and Southern blots were washed with sodium saline citrate (SSC), sodium dodecyl sulfate (SDS) at 60°C and exposed with BioMax MS films and Transcreen HE (Kodak).

### *clag9* Knockout

*P. falciparum* 3D7 genomic DNA was used to PCR amplify *clag9* exons 1 (bp 1 to 761) and 8 (bp 3652 to 4418) (Supplementary Table 1), the fragments were digested with SacII/SpeI and EcoRI/NcoI, and cloned into the vector pCC1 [30]. *P. falciparum* was transfected with this construct leading to the disruption of *clag9* by allelic exchange and insertion of the hDHFR selectable marker.

### Antibodies

Pooled human immune sera [31], anti-Pf332 mAb51-22 and rabbit purified IgG [32], anti-KAHRP mAb89 [33], anti-CD36 mAbFA6-152 [34], and mAb7H8/50 [35] have been described. Anti-Gluthathione S-transferase (GST) mAb clone GST-2 (Sigma), anti-hemagglutinin A mAb16B12 (Abcam), fluorochrome-conjugated secondary antibodies (Molecular Probes), and guinea pig anti-ATS serum (Charles Rivers Laboratories, France) were also used.

### Indirect Immunofluorescence Assay (IFA)

Immunofluorescence assays were done as previously described [32]. Surface immunofluoresence assays of D10 and FCR3^CD36^ were performed on trypsin-treated PRBC using pooled human immune sera (1∶5) followed by anti-human Alexa Fluor 488 (1∶100). Nuclei were stained with 0.2 mg/ml 4′,6-diamidino-2-phenylindole (DAPI; Molecular Probes) and 10 µg/ml propidium iodide (Sigma) to confirm cell viability. Parasites were visualized by confocal fluorescence microscopy (Nikon Eclipse TE2000-E), the images were captured with a Nikon D-Eclipse C1 camera and Nikon EZ-C1 software version 3.80.

### Iodination and Immunoprecipitation

Plasmion-enriched PRBC were labelled with ^125^I before incubation with different concentrations of trypsin (0, 10, or 100 µg/ml) or chymotrypsin (100 µg/ml) as described [36]. The Triton-X100 insoluble-SDS soluble (TX100_ins_-SDS_sol_) fractions were immunoprecipitated with a pool of human immune sera [31].

### Western blot

Western blots were performed as described [32]. TX100_ins_-SDS_sol_ extracts from trypsin-treated mature parasites were reacted with anti-ATS serum (1∶400) followed by alkaline phosphatase-conjugated anti-guinea pig secondary antibodies (1∶2500) and developed with NBT/BCIP (Promega). The *clag9* knockout clones KOE5 and KOF4 were similarly analysed using rabbit anti-CLAG9 antibodies (1∶1000) [37] and revealed with SuperSignal West Pico Chemiluminescent Substrate (Pierce, Thermo Scientific).

### CIDR1α binding analysis

The M2 CIDR1α domain (minimal CD36 binding site) [38] from the following *var* genes: D10*^var1^* (this work), FCR3*^var1CSA^* [36], and MC*^varCD36^* [38] were cloned into the pDisplay vector (Invitrogen) (Supplementary Table 1) and expressed on the surface of COS-7 cells [39]. CD36 binding assays were performed as described [36], [39] using magnetic protein A-conjugated Dynal beads (Invitrogen) and mAbFA6-152. The efficiency of COS-7 transfection was verified by detection of the pDisplay HA tag by IFA with mAb16B12 followed by anti-mouse Alexa Fluor 488.

### Cytoadherence assay

Assays were performed as described [36] with 10 µl of purified receptors immobilized on plastic Petri dishes: 10 µg/ml CD36 and ICAM-1 (R&D systems), 5 µg/ml gC1qR [40], 1 mg/ml CSA and CSC (Sigma). Control spots were coated with 1% BSA (fraction V, Sigma). FCR3 panned on CD36 (FCR3^CD36^) was used as positive control.

### Atomic Force Microscopy (AFM)

Plasmion-selected trophozoites and schizonts were fixed in 4% paraformaldehyde and 0.1% glutaraldehyde for 30 min on ice and washed twice with PBS. PRBC were immobilized on glass bottom WillCo dishes (WillCo Wells) activated using a low-pressure plasma 0_2_ (Femto, Diener Electronic) [41], [42]. The dishes were then covered with poly-L-lysine (Sigma) for 1 hour and rinsed with water. Experiments were conducted at room temperature on a stand alone AFM (Bioscope 2, Veeco Instruments) equipped with a Nonoscope V controller, mounted on an optical inverted microscope (Axiovert 200 M, Zeiss). Bright-field video images were acquired with a CoolSnap_ES_ camera (Roper Scientific) driven under MetaMorph software (Molecular Devices). AFM images were acquired in Tapping Mode™ using Veeco's silicon RTESP Tip (radius of curvature <10 nm), plane-fitted (1^st^ order), and processed under Vision software to get 3D representation. Height profiles of typical knobs were acquired with Veeco's integrated software and plotted using R 2.6.1. For each parasite strain three images showing the greatest dispersion in knobs sizes were selected.

### Statistical Analysis

Data were analysed with Minitab® 15 using a 1-way analysis of variance (ANOVA), including Tukey's test for multiple comparisons.

## Results

### *P. falciparum* D10 binding assays

**Figure 1:**
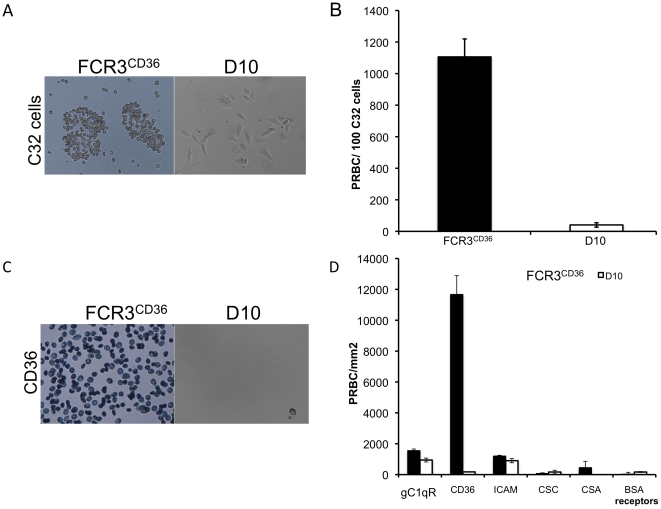
Binding assay on C32 melanoma cells. **A.** *P. falciparum* D10 mature stages are unable to bind to expressed receptors of C32 melanoma cells (CD36≫ICAM-1≫CSA) compared to the positive control FCR3^CD36^. **B.** The graph illustrates the number of bound PRBC per 100 C32 cells for FCR3^CD36^ (black) and D10 (white). Results are the means ± standard deviation from five experiments. **C.** Microscopic images of Giemsa stained FCR3^CD36^ and D10 on purified receptors. **D.** Quantification of binding assays for FCR3^CD36^ (black) and D10 (white) on purified receptors spotted on plastic Petri dishes. Results are the means ± standard deviation from five experiments.

Previous studies have shown that *P. falciparum* bulk-cultured parasites can be enriched for their binding to specific endothelial cell receptors [43]. C32 melanoma cells mainly express the CD36 receptor on their surface, and in lesser amounts the receptors ICAM-1 and CSA [44], [45]. To determine the adhesive properties of *P. falciparum* D10 PRBC, we performed binding assays on C32 cells [25]. Asynchronous cultures of *P. falciparum* D10 and FCR3^CD36^ were incubated with C32 cells, carefully washed to remove unbound PRBC, and the number of adherent PRBC determined. We observed that D10 PRBC did not bind to the melanoma cells, whereas FCR3^CD36^-infected erythrocytes were seen to adhere (Fig. 1A and B). Furthermore, no D10 adhesion was observed (data not shown) to human umbilical vein endothelial cells (HUVEC), a line to which some non-cytoadherent parasites are able to bind [46].

As several adhesins are implicated in the cytoadherence of PRBC, we further tested the binding of PRBC on purified CD36, ICAM-1, and some receptors rarely expressed in *in vitro* culture such as CSA and gC1qR. We did not observe any detectable binding of D10 to the tested receptors, which is in clear contrast to FCR3^CD36^ control adherence experiments (Fig. 1C and D). Repeated panning assays (3 times) on C32 cells and CD36 receptor did not yield any increase in adherence supporting the idea that D10 has irreversibly lost the adhesion phenotype.

### Characterization of the *P. falciparum* D10 variant PfEMP1

**Figure 2:**
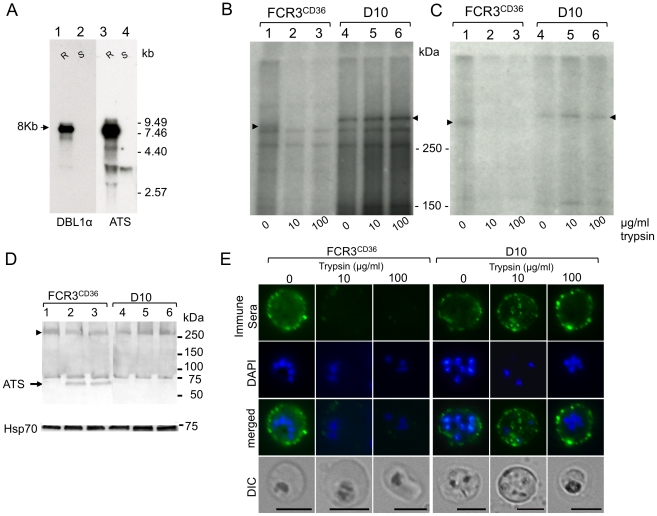
Analysis of *P. falciparum* D10. **A.** Northern blot. Total RNA (10 µg) from *P. falciparum* D10 intra-erythrocytic stages hybridized with the D10 DBL1αdomain of PfEMP1 probe (lanes 1–2), the expected 8 kb transcript was detected. The same membrane was hybridized with the ATS probe to control for loading (lanes 3–4). R: Ring stages (6–12 h after invasion), S: Schizonts (34–36 h after invasion). Size markers are in kb. **B–D.** Effect of trypsin on PfEMP1 of FCR3^CD36^ and D10 strains. **B.** Surface iodination. SDS-PAGE analysis of ^125^I-labelled FCR3^CD36^ (lanes 1–3) and D10 (lanes 4–6). **C.** Immunoprecipitation of FCR3^CD36^ (lanes 1–3) and D10 (lanes 4–6) TX100_ins_-SDS_sol_ trypsin-treated extracts with a pool of human immune sera. Arrowheads indicate the PfEMP1 antigen. Molecular weight markers are in kDa. **D.** Western blot. Mature FCR3^CD36^ and D10 parasites were treated with 0 µg/ml (lanes 1 and 3), 10 µg/ml (lanes 2 and 4), or 100 µg/ml trypsin (lanes 3 and 6). TX100_ins_-SDS_sol_ extracts were probed with goat anti-ATS and alkaline phosphatase-conjugated anti-goat and revealed with NBT/BCIP. The ATS antigen is shown by an arrow and an arrowhead indicates full- length PfEMP1. The same membrane was reacted with Hsp70 antibodies to control for loading. **E.** Surface immunofluorescence of FCR3^CD36^ and D10. Plasmion-selected parasites were treated with trypsin prior to incubation with pooled human immune sera (PIAG) followed by goat anti-human IgG Alexa Fluor 488. Scale bars: 5 µm.

RT-PCR analysis using primers to amplify the conserved DBL1α domain indicated that a single type of *var* gene is transcribed by ring stage D10 parasites [47]. Northern blot analysis using a probe corresponding to the DBL1α domain of this *var* gene showed a unique major 8 kb transcript in ring stages (Fig. 2A, lane 1). This transcript was not observed in D10 trophozoites and schizonts (Fig. 2A, lane 2) or in any FCR3^CD36^ blood stages (data not shown). Successive hybridization with a conserved ATS probe confirmed that a single major transcript is present in ring stages of D10 (Fig. 2A, lanes 3, 4).

Next, we verified whether the *var* transcript was translated into the protein PfEMP1 and expressed on the surface of the PRBC. Surface iodination experiments followed by immunoprecipitation with a pool of human immune sera showed that both FCR3^CD36^ and D10 displayed a protein of apparent high molecular weight of approximately 300 kDa present in the TX100_ins_-SDS_sol_ fraction. Whereas the positive control FCR3^CD36^ was readily digested by 10 µg/ml of trypsin (Fig. 2B and C, lanes 1–3), the PfEMP1 expressed by D10 was quite insensitive to digestion with even 100 µg/ml of trypsin (Fig. 2B and C, lanes 4–6) or 100 µg/ml of chymotrypsin (data not shown). A ∼75 kDa fragment corresponding to the intracellular domain of PfEMP1 was detected by Western blotting exclusively in the FCR3^CD36^ trypsin-treated extracts reacted with anti-ATS antibodies (Fig. 2D lane 1–3). In contrast, these antibodies reacted with the full-length PfEMP1 of D10 treated extracts (Fig. 2D lane 4–6).

PfEMP1 localization was also confirmed by surface IFA of live PRBC following trypsin treatment. As predicted surface labelling was sensitive to trypsin in FCR3 and resistant in D10 parasites (Fig. 2E). Furthermore, cell integrity was verified by propidium iodide stanning (data not shown). These findings indicated that a trypsin-resistant PfEMP1 is expressed on the surface of D10 that has lost its adhesive properties.

### Protein trafficking by *P. falciparum* D10

**Figure 3:**
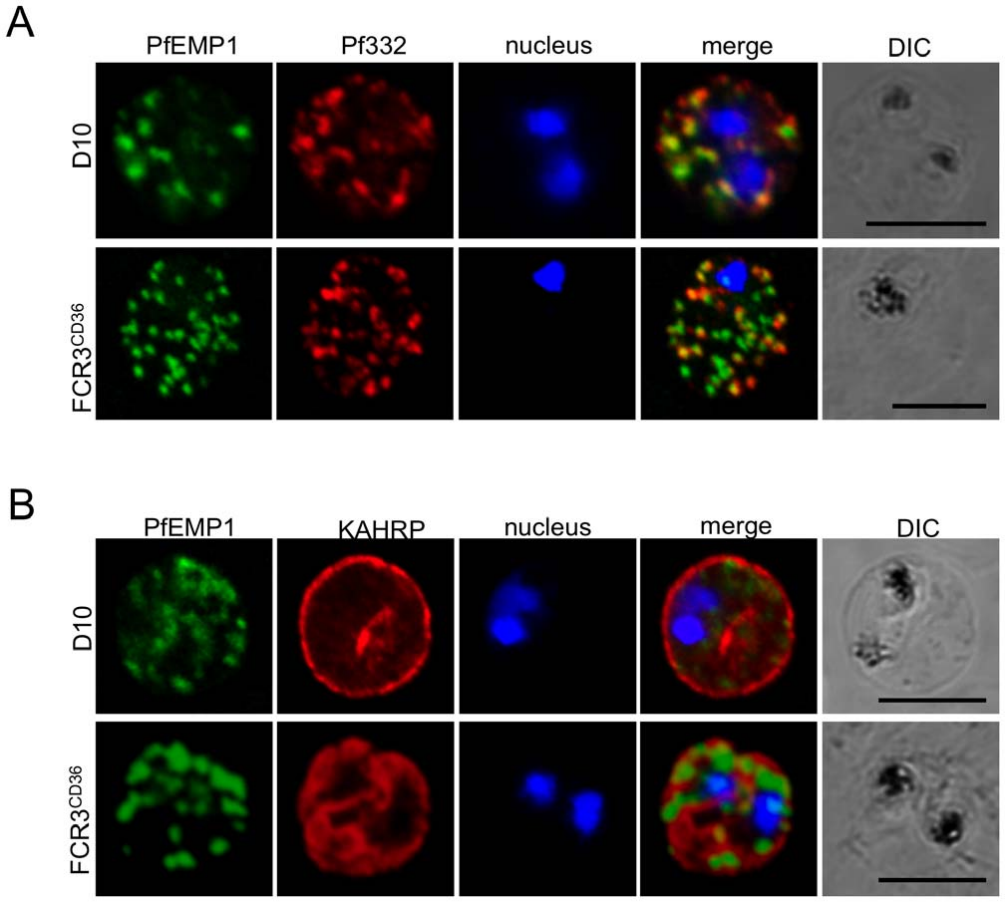
Localization of parasite exported proteins. **A.** Double-labelling of synchronous air-dried D10 (top row) and FCR3^CD36^ (bottom row) with guinea pig anti-PfEMP1 antibodies (1∶500) and purified rabbit anti-Pf332 IgG (1∶200) revealed with a mixture of goat anti-guinea pig Alexa Fluor 488 (1∶500) and Alexa Fluor 594-conjugated anti-rabbit IgG (1∶1000). **B.** Labelling of PfEMP1 (1∶500, green) and knobs on the PRBC surface with monoclonal anti-KAHRP (mAb89, 1∶800, red), followed by anti-guinea pig Alexa Fluor 488 and anti-mouse Alexa Fluor 594. Scale bars: 5 µm.

To further investigate the non-binding phenotype we analyzed by immunofluorescence whether intracellular protein trafficking was affected in D10 parasites. Several *P. falciparum* polypeptides are exported through the red blood cell cytoplasm in association with the Maurer's clefts, and these may be essential for the correct assembly of the cytoadherence complex at the PRBC surface [48]. PRBC double-labelled with the Maurer's clefts marker Pf332 and PfEMP1 antibodies (Fig. 3A), showed that PfEMP1 was associated with the Maurer's clefts in transit through the RBC cytoplasm. Comparable results were obtained with the FCR3^CD36^ PRBC (Fig. 3A), however, the D10 Maurer's clefts labelling detected with anti-Pf332 appeared more diffuse than in FCR3^CD36^ suggesting a different morphology. A similar altered Maurer's clefts architecture was observed in REX1 mutant parasites [49], one of the 25 genes lost in the chromosome 9 deletion of D10.

Furthermore, mAb89 reacted with knobs at the RBC membrane of D10-infected erythrocytes indicating that KAHRP, which interacts with the PfEMP1 ATS region, is correctly addressed to the RBC membrane (Fig. 3B). These data suggest that trafficking and export of the studied marker proteins and PfEMP1 are apparently not affected in D10 parasites.

**Figure 4:**
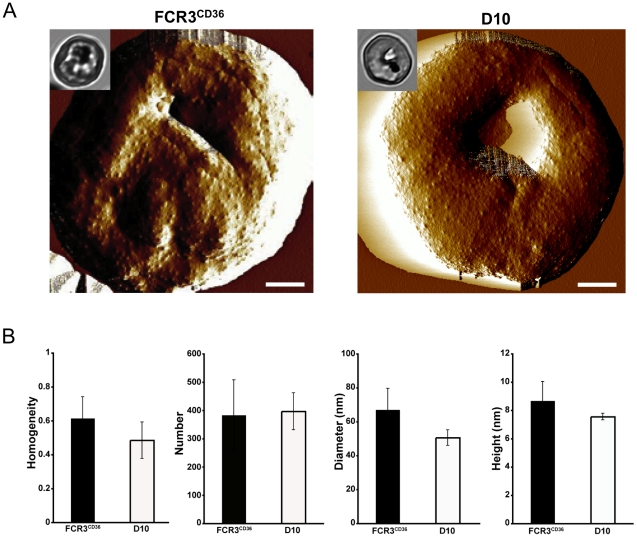
AFM analysis of erythrocytes infected by *P. falciparum* FCR3^CD36^ and D10. **A.** Representative amplitude images of infected red blood cells. Insets show Giemsa staining of the analysed PRBC. **B.** Height images were used to measure knob homogeneity, number, diameter and height on infected erythrocytes. Knob homogeneity and number were analyzed using mean ± standard deviation (5≤N cells≤15). Knob diameter and height were analyzed using mean ± standard error of the mean (5≤N cells≤15). Scale bars: 1 µm.

Because PfEMP1 is anchored to the cytoskeleton of the infected erythrocyte through interactions with the knob component KAHRP, we verified if the knobs from D10 had a structure similar to parasites expressing functional PfEMP1 on their surface. The visualisation by AFM of the PRBC revealed only subtle changes in the knob morphology between D10 and FCR3^CD36^ (Fig. 4A). No difference was observed in the amount of knobs and their distribution on the erythrocyte surface for these strains. The diameter and height of D10 knobs were slightly smaller than that from FCR3^CD36^ (Fig. 4A and B). These results imply that although the knob morphology is marginally affected by the chromosome 9 deletion it is not causing the loss of cytoadherence of D10 parasites.

### The CIDR1α domain of the PfEMP1 expressed in D10 binds to CD36

**Figure 5:**
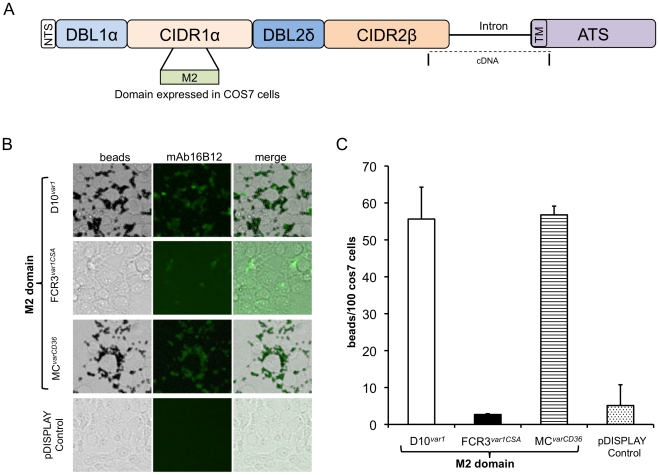
Expression and binding of D10*^var1^* CIDR1α minimal domain. **A.** Diagram of *D10^var1^*. The different domains are shown, including the location of the PfEMP1 CIDR1α minimal domain (M2) used for expression by COS-7 cells. The region spanning the intron between exons 1 and 2 of D10*^var1^* was sequenced from cDNA. **B.** Beads bound to COS-7 cells expressing M2 on the surface. **C.** Quantification of binding on COS-7 cells. The CIDR1α minimal domain of the D10*^var1^* PfEMP1 binds equally well to COS-7 cells as that of the Malayan Camp (MC*^varCD36^*, positive control). In contrast, there is no significant binding of the FCR3*^var1CSA^* M2 domain. pDisplay control represents COS-7 cells transfected with pDisplay not containing the insert.

We cloned and sequenced the entire *var* gene transcribed in D10 (called *D10^var1^*) and determined that the open reading frame of this transcript is composed of NTS-DBL1α-CIDR1α-DBL2δ-CIDR2β-TM-ATS domains (Fig. 5A). The different domains of PfEMP1 can be classed according to their binding regions allowing the prediction of the corresponding adhesion receptors [5], [39]. The M2 minimal domain from PfEMP1 CIDR1α is necessary and sufficient for binding to the CD36 receptor [38]. Thus, to better characterize *D10^var1^*, the HA tagged M2 domain was expressed on the surface of COS-7 cells (Fig. 5A). Cells were incubated with recombinant CD36 and M2-CD36 surface complexes we detected using anti-CD36 mAb-coated Dynal beads. COS-7 cells surface-expressing the M2 domain from D10*^var1^* and the positive control MC*^varCD36^* are shown in Fig. 5B. No significant differences (*P* = 0.961) in binding were observed. In contrast, COS-7 cells expressing M2 domain from the negative control FCR3*^var1CSA^*, showed significantly less coated beads on the surface (*P*<0.001) (Fig. 5B and C). Transfected COS-7 cells expressing M2 domains were visualized after the binding assay by IFA staining of the HA-domain fused to M2 (green fluorescence). Our results demonstrate that the M2 domain of *D10^var1^* is functional when expressed outside the D10 infected erythrocyte context and indicate that D10 PRBC have lost their capacity for functional expression of parasite-mediated adhesion, maybe due either to a conformational defect, improper insertion into the RBC membrane, or missing post-translational modifications of PfEMP1.

### CLAG9 is not essential for binding to CD36

**Figure 6:**
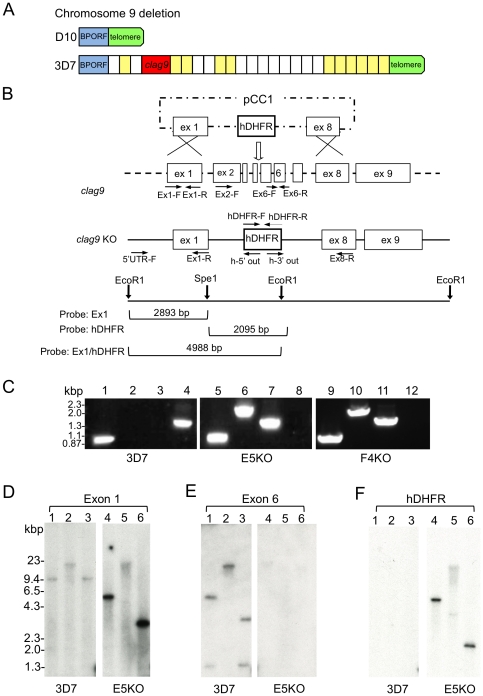
Generation and characterization of *clag9* KO. **A.** Subtelomeric chromosome 9 deletion in D10. Schematic representation of the genes missing from the right arm of chromosome 9, as compared to 3D7. The break point open reading frame (*BPORF*) is present but none of the 25 genes between the *BPORF* and the telemores, including *clag9* (red), were detected by PCR (data not shown). Each block corresponds to a single gene, *clag9* and *BPORF* are larger for emphasis and the size of blocks does not illustrate gene size. The genes shown in yellow have been functionally identified. The identity of the remaining genes has not yet been elucidated. **B.** Plasmid pCC1 exon 1+8 knockout leading to the disruption of the *clag9* gene by allelic exchange with insertion of the hDHFR selection marker. Primers (arrows) used for verification of KO and hDHFR integration, the restriction sites, and Southern blot expected fragment sizes are shown. **C.** PCR analysis of 3D7 (lanes 1–4), the *clag9* E5KO (lanes 5–8), and F4KO (lanes 9–12). Lanes 1, 5, and 9: 5′UTR-F - exon 1-R; lanes 2, 6, and 10: 5′UTR-F- hDHFR 5′ out; lanes 3, 7, and 11: hDHFR 3′ out – exon 8-RC3′; lanes 4, 8, and 12: exon 2-F - exon 6-R. **D–F.** Southern blot of *clag9* knockout. Genomic DNA digested by EcoRI (lanes 1 and 4), SpeI (lanes 2 and 5) or EcoRI+SpeI (lanes 3 and 6) separated on 0.8% agarose gel, transferred onto Hybond N+ and hybridized with different probes. **D.** Exon 1, present in both 3D7 and the *clag9* E5KO. **E.** Hybridization of Exon 6 only with 3D7. **F.** Integration of the hDHFR selectable marker in the E5KO.

**Figure 7:**
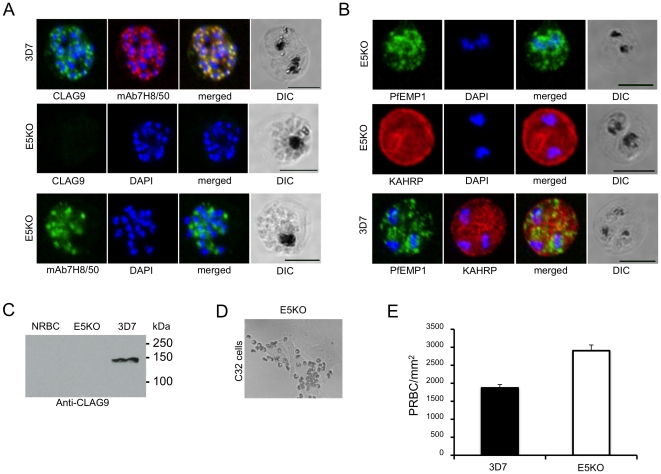
Immunofluorescence and binding assays of *clag9* E5KO. **A.** Labelling of CLAG9 in 3D7 and E5KO. Note there is labelling of E5KO rhoptries with mAb7H8/50 but not with anti-CLAG9 specific sera due to gene disruption. Scale bars = 5 µm. **B.** Trafficking of PfEMP1 and KAHRP by E5KO; labelling was identical to 3D7. Scale bars = 5 µm. **C.** Chemiluminescent western blot analysis of normal red blood cells (NRBC), E5KO and 3D7 parasite extracts reacted with anti-CLAG9 antibodies. **D.** Binding of *clag9* E5KO on C32 melanoma cells. **E.** Binding assay of 3D7 and E5KO PRBC on purified CD36 receptor. Error bars indicate the standard error of the mean from 4 experiments.

The site of the chromosome breakpoint leading to the deletion of the subtelomeric region was determined by PCR on the telomere repeats and the genes suspected to be located there. Based on the 3D7 genome sequence D10 has deleted around 120 kbp of the right arm of chromosome 9 [50] representing 25 genes, including *clag9* (Fig. 6A), encoding a rhoptry protein [37]. CLAG9 was initially implicated in parasite adhesion to CD36 and C32 cells [16] and recently in the adhesion to placental CSA [15]. To determine its potential role in the cytoadherence phenotype, we inactivated the *clag9* gene from *P. falciparum* 3D7. Targeted disruption of *clag9* was performed by replacing exons 2 through 6 with the hDHRF selectable marker (Fig. 6B). Gene knockout and integration of the hDHFR cassette were confirmed by PCR analysis (Fig. 6C) and Southern blotting using probes for exons 6 and hDHFR (Fig. 6D–F). CLAG9 was not detected in two knockout clones (E5KO and F4KO), either by immunofluorescence (Fig. 7A and B) or western blotting (Fig. 7C). In contrast to earlier reports implicating CLAG9 in cytoadhesion, our KO parasites bound to both C32 cells (Fig. 7D) and CD36 (Fig. 7E) at levels comparable to our control 3D7 parasites. These results show that the *clag9* gene product is not directly implicated in cytoadhesion to CD36 and, therefore, in the non-cytoadherent phenotype of the D10 strain.

## Discussion

*P. falciparum* export several proteins beyond the parasite membrane to the erythrocyte surface assuring the parasite's survival within the human host. A large number of these exported molecules have been described that are essential for, or contribute to, the trafficking of the major virulence factor PfEMP1 to the erythrocyte surface. Here we describe a novel adhesion mutant phenotype. In D10 PfEMP1 is trafficked to the surface of PRBC but is unable to bind to host adhesion receptors. Even repeated panning experiments on C32 cells or recombinant CD36 bound to plastic dishes did not yield any increase in binding, showing that no other *var* gene is able to restore the adhesion phenotype. Bulk-cultured parasites typically contain minor populations expressing a number of different *var* gene members (including CD36-binders). Thus, our data indicate that in D10 non-functional PfEMP1 surface expression is implicated in the irreversible loss of cytoadherence. We also have characterized another non-cytoadherent strain, T9-96 [46] with similar results (data not shown), suggesting that this may be a common adhesion phenotype linked to chromosome 9 deletion.

Trypsin and chymotrypsin digestion experiments of surface iodinated PRBC proteins suggest that PfEMP1 molecules expressed at the surface of D10 display an unusual conformation that protect them from the predicted protease cleavage. It may also be the reason for the loss of the expected binding to CD36. This idea is supported by the fact that recombinant CIDR1αof *D10^var1^* binds efficiently to CD36 when expressed by another eukaryotic cell. PfEMP1 may need ‘helper’ proteins acting as a support or chaperones to assume the right shape to function as a ligand. Alternatively, post-translational modifications and/or processing of the protein during export may be needed to obtain functional adhesion molecules. Further studies are required to explore this hypothesis.

Are there candidate genes deleted on chromosome 9 of D10 that could contribute to the described adherence phenotype of D10? Several studies have implicated *clag9* in parasite adhesion to CD36 and CSA [15], [16]. We revisited this question by irreversibly disrupting the *clag9* gene by allelic replacement. The *clag9* knockout in 3D7 resulted initially in low CD36 binding parasites, which can be explained by the time taken to obtain cloned knockout lines. However, after one panning step on CD36, these *clag9* mutants gained an adhesion capacity similar to the reference parasite 3D7. Thus, our data demonstrate that 3D7, in contrast to previous studies, does not depend on *clag9* for the PfEMP1 adhesion process. At this stage, it is difficult to explain the different set of *clag9* knockout data. It cannot be ruled out that other gene deletions known to occur during *in vitro* culture of 3D7, or associated with the gene deletion [13], may have been picked up at the cloning step after the knockout selection. This may mask the observed phenotype of *clag9* in the study reported by the Kemp laboratory [16].

*clag* genes form a small gene family (5 members) and two members (clag3.1 and 3.2) have recently been shown to integrate into the erythrocyte membrane and induce a new permeation pathway [51]. It is tempting to speculate that CLAG9 may accomplish a similar function, a role that is not essential during *in vitro* culture. Since CLAG9 apparently integrates into the erythrocyte membrane, it may interact with a particular member of the adhesion molecule family as has recently been suggested for var2CSA [15]. Further receptor binding studies of our *clag9* knockout are needed to fully explore this possibility.

Another candidate gene deleted on chromosome 9 is the Maurer's cleft-resident ring exported protein 1 (REX1). Its deletion results in alteration of Maurer's clefts architecture [49] and a reduction of cytoadhesion to CD36 [13]. These data point to REX1 as a plausible candidate gene for the observed adhesion phenotype in D10. We assume that additional co-deleted genes may act together with REX1 resulting in the complete loss of adhesion. Although D10 is not known to possess any of the REX proteins on chromosome 9, our data indicate that transit of proteins in this parasite strain appears functional. Indeed, labelling of PfEMP1 in D10 seems identical to that observed with the cytoadherent FCR3^CD36^. REX1 mutation resulted in the co-deletion of a subtelometic region of 3D7 chromosome 2 including KAHRP [13]. In our D10 parasites this is not observed since our antibodies label KAHRP and AFM demonstrates the presence of knobs. Dixon *et al.* [13] observe that REX1 deletion leads to trypsin-resistant PfEMP1 and conclude that no PfEMP1 is exposed on the PRBC. In the absence of surface iodination experiments, however, it cannot be excluded that trypsin-resistant PfEMP1 is expressed on the surface. Further characterization of REX1 may unravel the function of this protein in the adhesion process.

A large-scale loss-of-function study designed to characterize candidate genes involved in interactions with PfEMP1 or with a role in knob formation was performed by the Cowman laboratory [11]. Fifty genes were functionally analysed and a number of different adhesion phenotypes were linked to proteins required for loading of PfEMP1 to Maurer's clefts and function on the PRBC surface. However, this study did not include genes deleted on chromosome 9. In addition, the surface exposure of PfEMP1 was only analysed by trypsin treatment, which releases a small trypsin-resistant fragment (mainly composed of the ATS domain) between 70 and 90 kDa. Trypsin-resistant PfEMP1 was interpreted as being not exposed on the surface of PRBC. Our data using surface iodination experiments, however, show that parasites can expose non-functional trypsin-resistant PfEMP1. Since PfEMP1 molecules interact with KAHRP at the knob protrusions [9], we cannot rule out that the slightly modified knobs structure observed in D10 (see Fig. 4), could contribute to the complete loss of adhesion in D10. The role of the 25 genes deleted in D10 in this process will require further analysis. To answer these questions, work is in progress aiming to complement the deleted genes from chromosome 9 in D10 to establish which genes contribute to the cytoadherence phenotype.

In conclusion, we have identified mutant parasites with non-functional PfEMP1 surface expression. We also provide solid experimental evidence that CLAG9 does not contribute in the binding to CD36. In addition, our work illustrates that the factors leading to functional surface expression of PfEMP1 are not well elucidated and that the analysis of the genes deleted in D10 may contribute to our understanding of parasite virulence.

## Supporting Information

Table S1 Primers used to amplify the *P. falciparum D10^var1^* gene, PfEMP1 M2 minimal domain, *clag9* gene and *clag9* knockout.
(DOC)
