# Supplementary material for: clag9 Is Not Essential for PfEMP1 Surface Expression in Non-Cytoadherent Plasmodium falciparum Parasites with a Chromosome 9 Deletion
Source: PLoS One. 2011 Dec 19;6(12):e29039. doi: 10.1371/journal.pone.0029039 (PMC3242772; doi:10.1371/journal.pone.0029039)
Supplement: Table S1 — Primers used to amplify the P. falciparum D10var1 gene, PfEMP1 M2 minimal domain, clag9 gene and clag9 knockout. (DOC) [file pone.0029039.s001.doc]

**Supplementary Table 1**: Primers used to amplify the *P. falciparum* *D10^var1^* gene, PfEMP1 M2 minimal domain, *clag9* gene and *clag9* knockout

| Gene/Primes | Sequence 5’ - 3’ |
| --- | --- |
| **PfEMP1** |  |
| upsB | GTTAGAACATTTAAAATTATA |
| D10 all var | AGATATATGATAGATAATATAGATAGAGAG |
| D10DBL1α-F | GCCTGTGCTCCGTTTAGACG |
| DBL1α-2R | CCAGAACCATCTGTATGATG |
| D10DBL1α-R | ACATAATCGAAATTTGTAGGTAC |
| DBL1α-3F | ATAAGTGCAGAACAGAAACTTG |
| CIDR1α-F | TCAAAATGAAATATCAGGTGG |
| CIDR1α-R | ATCGTGTAGTTTCCC |
| DBL2δ-1F | GATAAAGACACGGAGGCACG |
| DBL2δ-2F | GGTGGTAGTATAACGGAGG |
| CIDR2β-1F | TGCGGGGGTTCTCCTAATGGAAAT |
| CIDR2β-2F | CTCTCCCCCGTTGAAGACGAAGAC |
| TM ATS-F | GGCGTCCTCCACCCTTGCCTGG |
| ATS-5F | CCCAAAGGAGAGTATGGAATGCCG |
| ATS-4R | CCTACCACGTCACATCATAATGTG |
| ATS-1F | GTTGCAAAAAATACAAATAGTGAT |
| D10end-R | AGATATTCCATATATCTGATATAGG |
|  |  |
| **pDisplay** |  |
| pDisplay 5' | AACCCACTGCTTACTGGCTTATCG |
| pDisplay 3' | AACAGATGGCTGGCAACTAGAAGG |
| pDisplayD10^var1^ 5’ | CCCCCCGGGGGTCAAAAGGTTACGTCT |
| pDisplayD10^var1^ 3’ | TCCCCGCGGCCTACTTCGGCACGAGC |
| pDisplayMC*^varCD36^* 5’ | CCCCCCGGGGAGGAAGACAAAATTATG |
| pDisplayMC*^varCD36^* 3’ | TCCCCGCGGGAGCGGGCGACACTTCT |
| pDisplayFCR3*^var1CSA^* 5’ | CCCCCCGGGGGGATAAATAGATGTAAACTG |
| pDisplayFCR3*^var1CSA^* 3’ | TCCCCGCGGGGATGTTTCACATGCTTCGTT |
|  |  |
| ***clag9*** |  |
| exon 1-F | CCCCCCGCGGATGATAATTTGGTTTATTCAACCAACGATTTTTTACAT |
| exon 1-R | CCCCACTAGTCCAATCATATTAAATATTCCTATATGATAGTAATTGGC |
| exon 8-F | CCCCGAATTCGAATTGGAAAAGCTAATTGATAATAAATTGATAGAGAAGC |
| exon 8-R | CCCCCCATGGACATCATCATTTCTTCTGTGAGAAAAATAGGTACCTTGTA |
|  |  |
| **clag9 KO** |  |
| 5'UTR-F | CATGTACCTTGTTATCTTTTATAC |
| exon 2-F | GGGCATTTTATTACTTTAAAATTAGC |
| exon 6-R | GCAAATCTTCAACACCCTTTC |
| exon 1-R | ACTAGTCCAATCATATTAAATATTCCTATATGATAGTAATTGGC |
| exon 8-RC3’ | CACATCATCATTTCTTCTGTGAGAAAAATAGG |
| hDHFR 5' out | CGATGCAGTTTAGCGAACCAACCAT |
| hDHFR 3’ out | CAAATTTGAAGTATATGAGAAGAATGATTAA |
